# Endoscopic Reverse Stapedotomy for Otosclerosis: A Technical Video Report

**DOI:** 10.7759/cureus.89914

**Published:** 2025-08-12

**Authors:** Ismail Nakkabi

**Affiliations:** 1 ENT Department, Hôpital Militaire Oued Eddahab, Agadir, MAR

**Keywords:** endoscopic ear surgery, otosclerosis, reverse stapedotomy, transcanal approach, virtual endoscopy

## Abstract

Endoscopic stapedotomy offers a panoramic transcanal view of middle-ear structures while minimizing canal trauma. Reverse stapedotomy, in which the footplate fenestration precedes the removal of the stapes superstructure, may improve footplate stability and procedural safety. The use of CT-based three-dimensional (3D) virtual endoscopy for surgical planning in otosclerosis remains uncommon. We present the case of a 35-year-old female with bilateral otosclerosis (stage 1B on high-resolution CT) and a 35 dB air-bone gap in the poorer-hearing ear. Preoperative CT-based 3D virtual endoscopy simulated the surgical view, revealing that a Rosen notch would be needed to visualize the stapes suprastructure. Endoscopic reverse stapedotomy was performed using a manual microtrephine for a 0.6 mm platinotomy, followed by superstructure removal and super-titanium piston placement. At six weeks, the tympanic membrane was intact, the prosthesis stable, and the air-bone gap (ABG) closed to within 10 dB.

This report highlights the value of integrating virtual endoscopy into stapes surgery planning to anticipate exposure limitations and guide targeted bony removal. The reverse sequence, combined with microtrephine fenestration, can be performed safely under endoscopic control. CT-based 3D virtual endoscopy can be a useful adjunct in planning endoscopic reverse stapedotomy, potentially optimizing surgical exposure and contributing to favorable anatomical and functional outcomes.

## Introduction

Otosclerosis is a common cause of conductive hearing loss in adults. Its surgical management typically involves stapedotomy with the placement of a prosthesis between the incus and the stapedial footplate. The endoscopic approach provides a panoramic view of the middle-ear structures while minimizing external canal trauma. Although preoperative imaging is routinely performed, the use of CT-based three-dimensional (3D) virtual endoscopy in otosclerosis surgery remains uncommon. This technology can enhance operative planning by anticipating line-of-sight constraints and guiding targeted exposure strategies. In this report, we describe an endoscopic reverse stapedotomy in which virtual endoscopy directly influenced the intraoperative decision to create a Rosen notch. We also present the favorable short-term postoperative anatomical and functional outcomes achieved with this approach.

## Technical report

A 35-year-old female patient presented with bilateral otosclerosis, classified radiologically as stage 1B on high-resolution CT. On the poorer-hearing side, pure-tone audiometry demonstrated a 35 dB air-bone gap (ABG), for which surgery was undertaken using a 4 K, 30-degree rigid endoscope.

**Figure 1:**
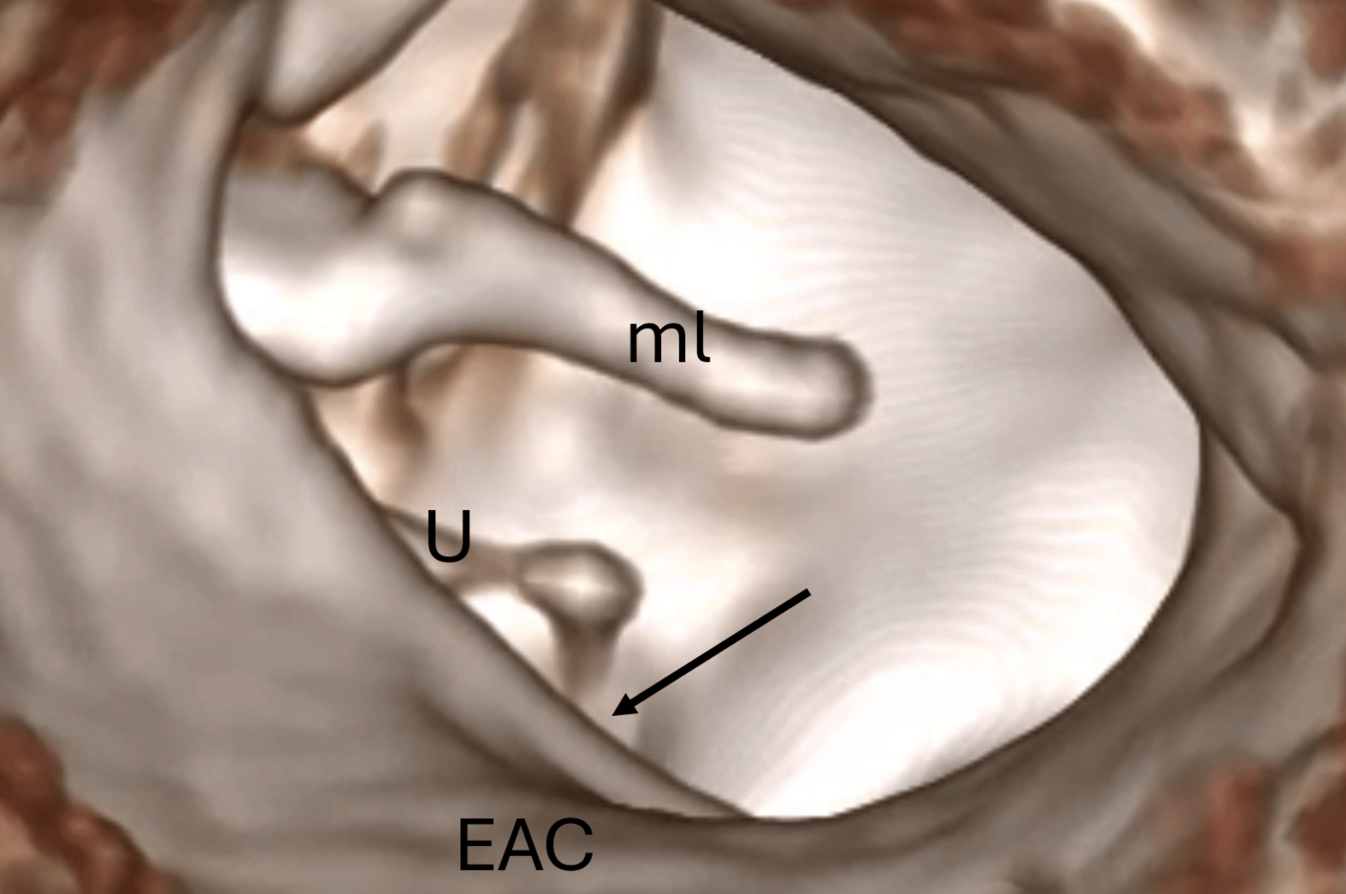
3D virtual endoscopic view based on CT scan CT: computed tomography; EAC: external auditory canal; ml: malleus; U: uncus

The surgical video (Video 1) begins with axial and coronal CT slices, followed by a 3D virtual endoscopic reconstruction of the external auditory canal and middle-ear space (Figure 1). This simulation confirmed that direct visualization of the stapes suprastructure would be restricted without limited posterior bony removal and prompted the decision to create a Rosen notch to obtain an unobstructed working corridor.

**Video 1:** Endoscopic reverse stapedotomy

**Figure 2:**
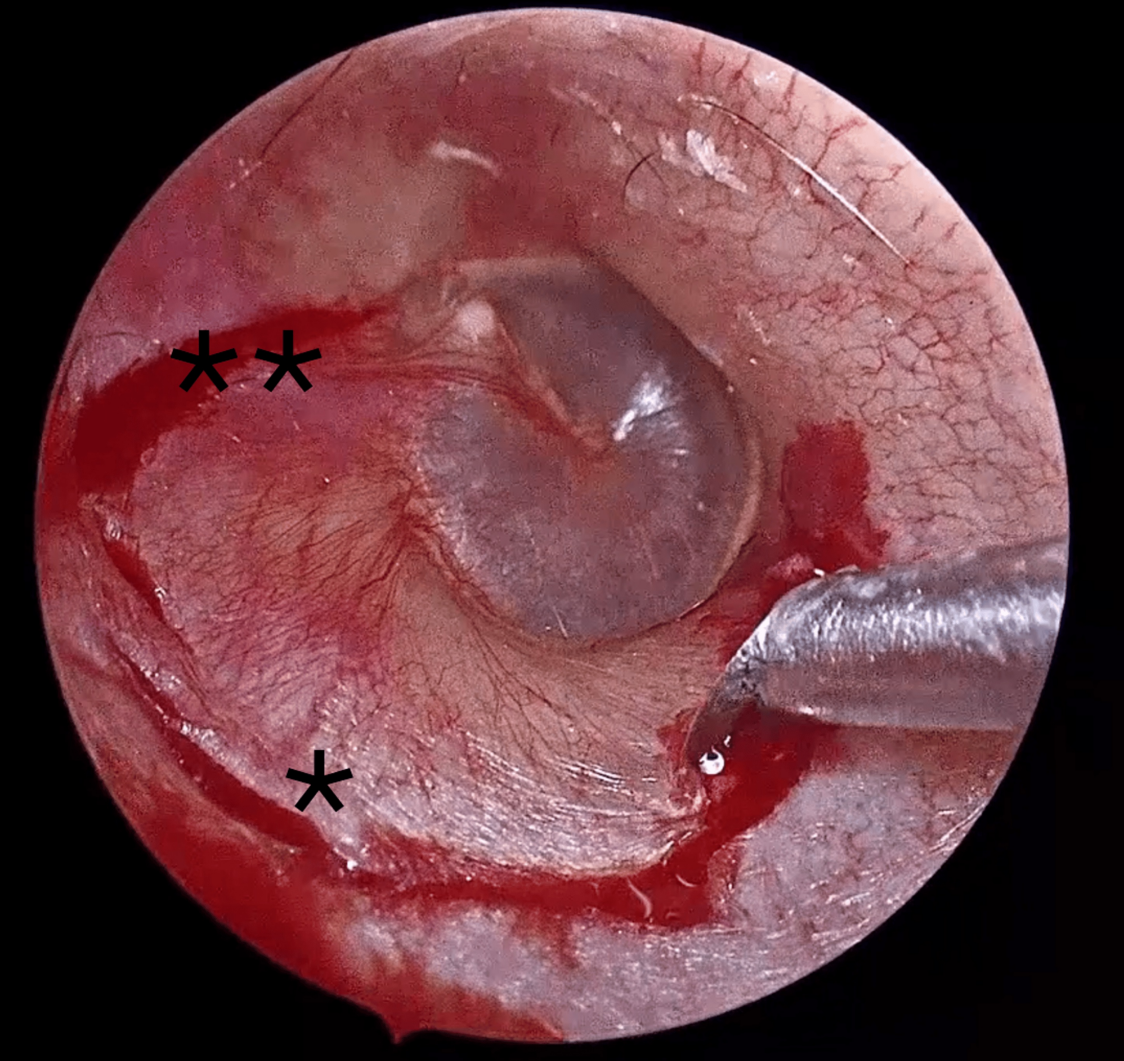
Incision (*) and counter-incisions (**) of the tympanomeatal flap

Intraoperatively, the tympanic membrane appeared normal. A tympanomeatal flap was elevated by making two counter-incisions -superior and inferior - in the external auditory canal skin with a Plester instrument, then joining them using a Rosen suction dissector (Figure 2). The flap was carefully elevated to expose the tympanic annulus, which was detached to enter the middle ear.

**Figure 3:**
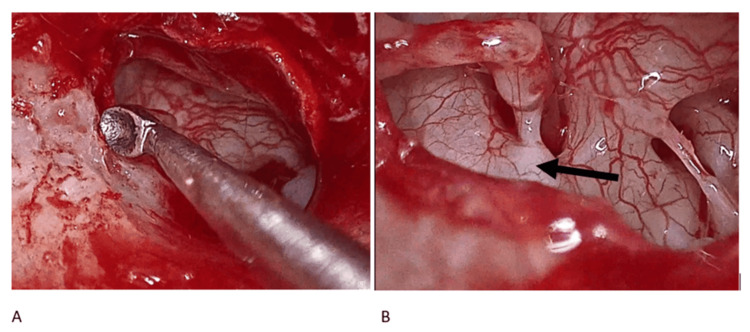
Visualization of the stapes superstructure A. Before performing the Rosen notch. B. After the notch is made. Note the visibility of the stapes pyramid (black arrow)

On entering the tympanic cavity, a characteristic hypervascularization of the promontory was noted, consistent with active otosclerosis. Ossicular mobility testing revealed normal malleus-incus motion but no stapes movement, confirming stapes fixation. As predicted by the preoperative virtual endoscopy, the stapes suprastructure was partially obscured. A Rosen notch was therefore created in the posterior canal wall using a microcurette while preserving the chorda tympani (Figure 3). This maneuver provided a direct view of the pyramidal eminence and allowed the use of straight instruments toward the stapes.

**Figure 4:**
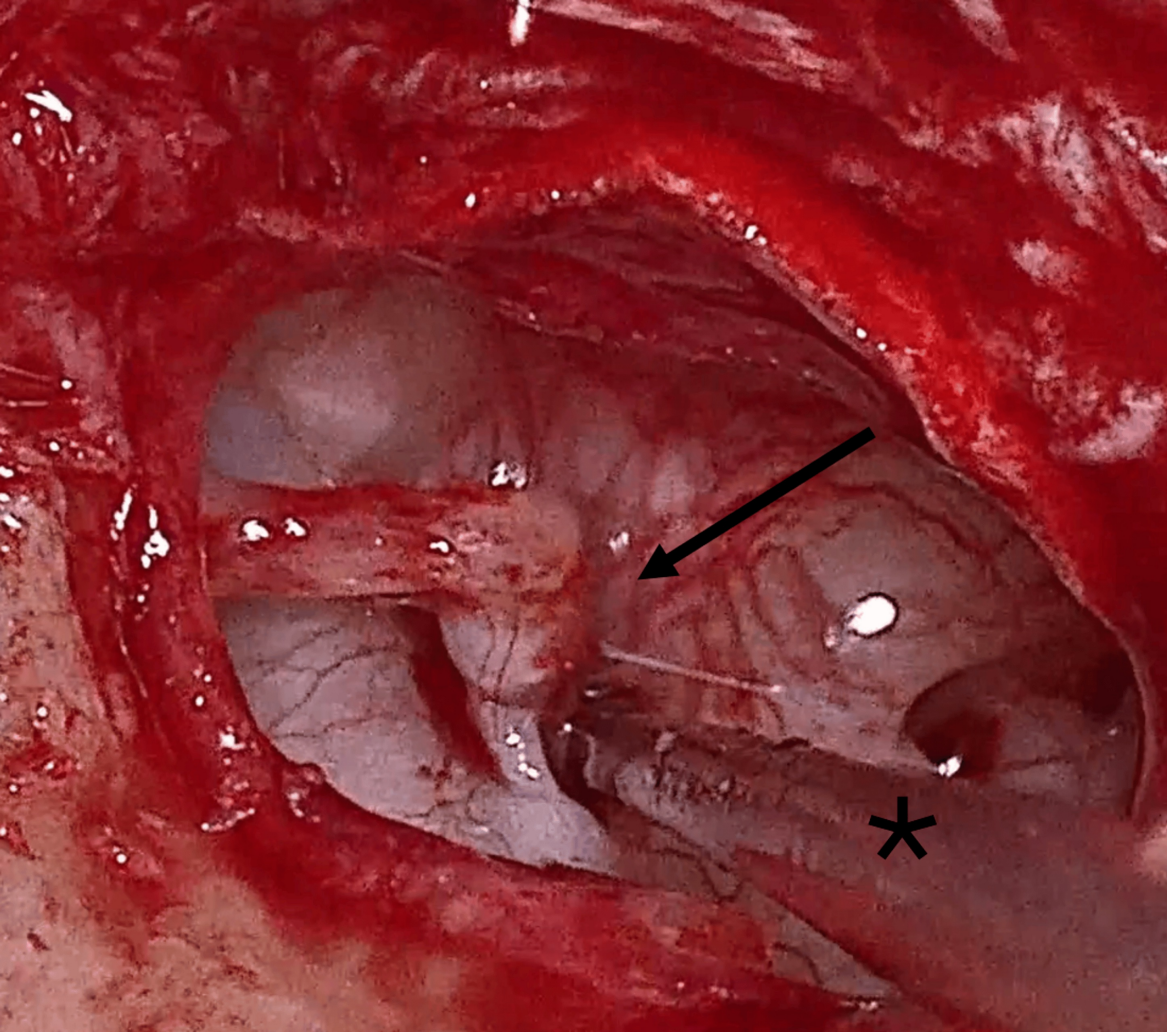
Microtrephine (*)-assisted stapedotomy with intact stapes superstructure (black arrow)

**Figure 5:**
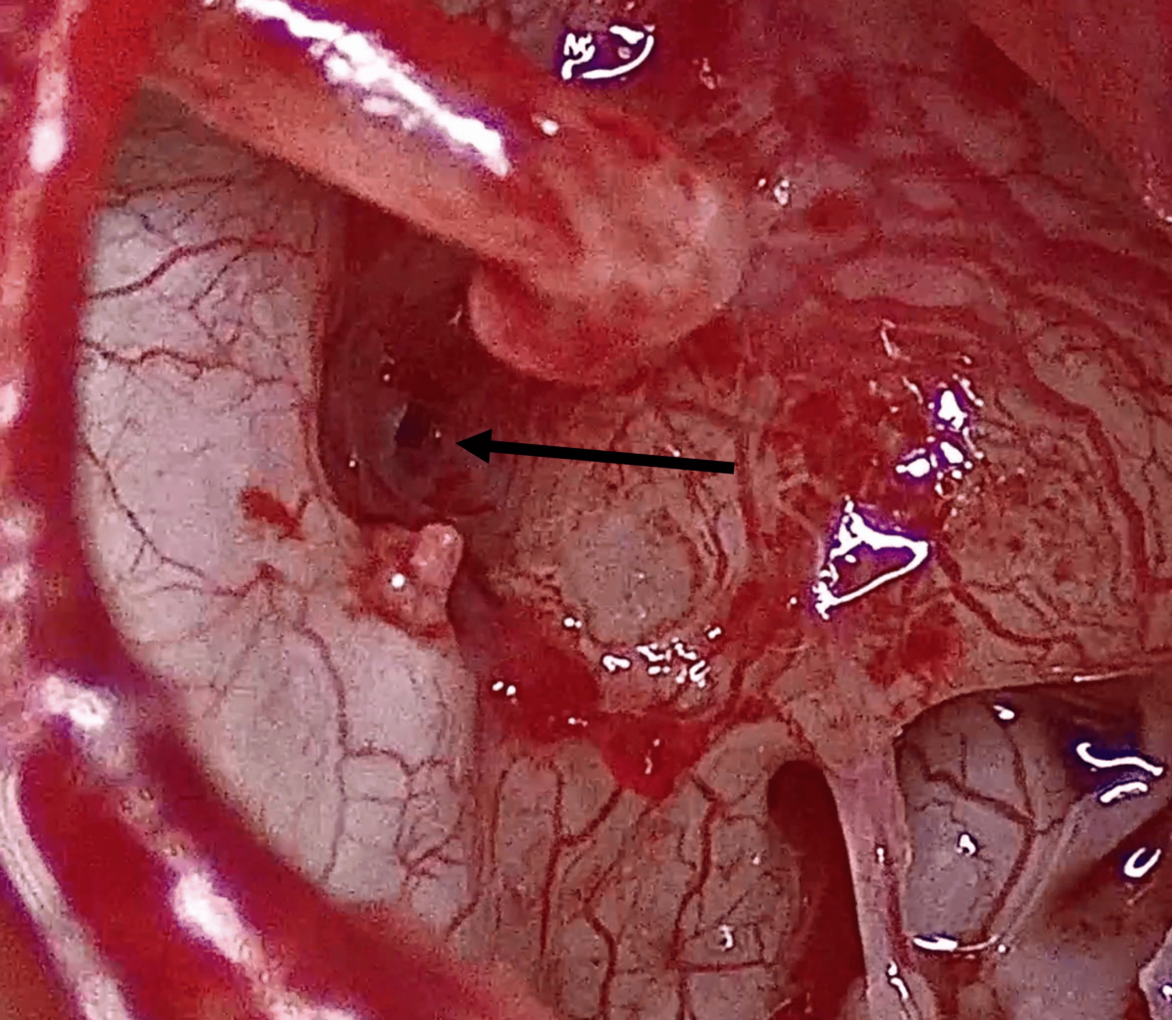
Posterior footplate platinotomy (black arrow)

A calibrated 0.6 mm platinotomy was performed (Figure 4) using a manual microtrephine, with the stapes suprastructure left in place to stabilize the footplate and reduce the risk of inward displacement. The incudostapedial joint was then separated, the stapedial tendon divided, and the stapes suprastructure removed, revealing the fenestrated footplate (Figure 5).

**Figure 6:**
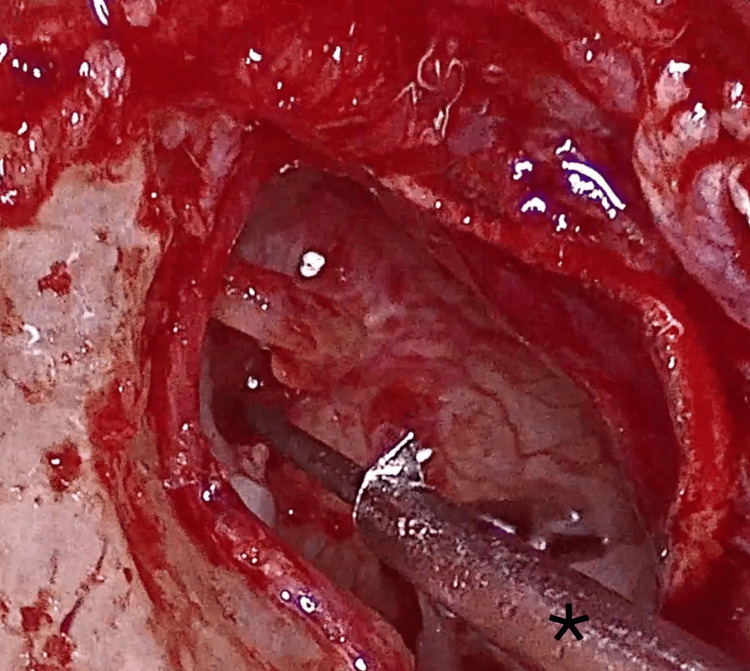
Measured distance (*, 4.5 mm) between the incus long process and the stapes footplate

**Figure 7:**
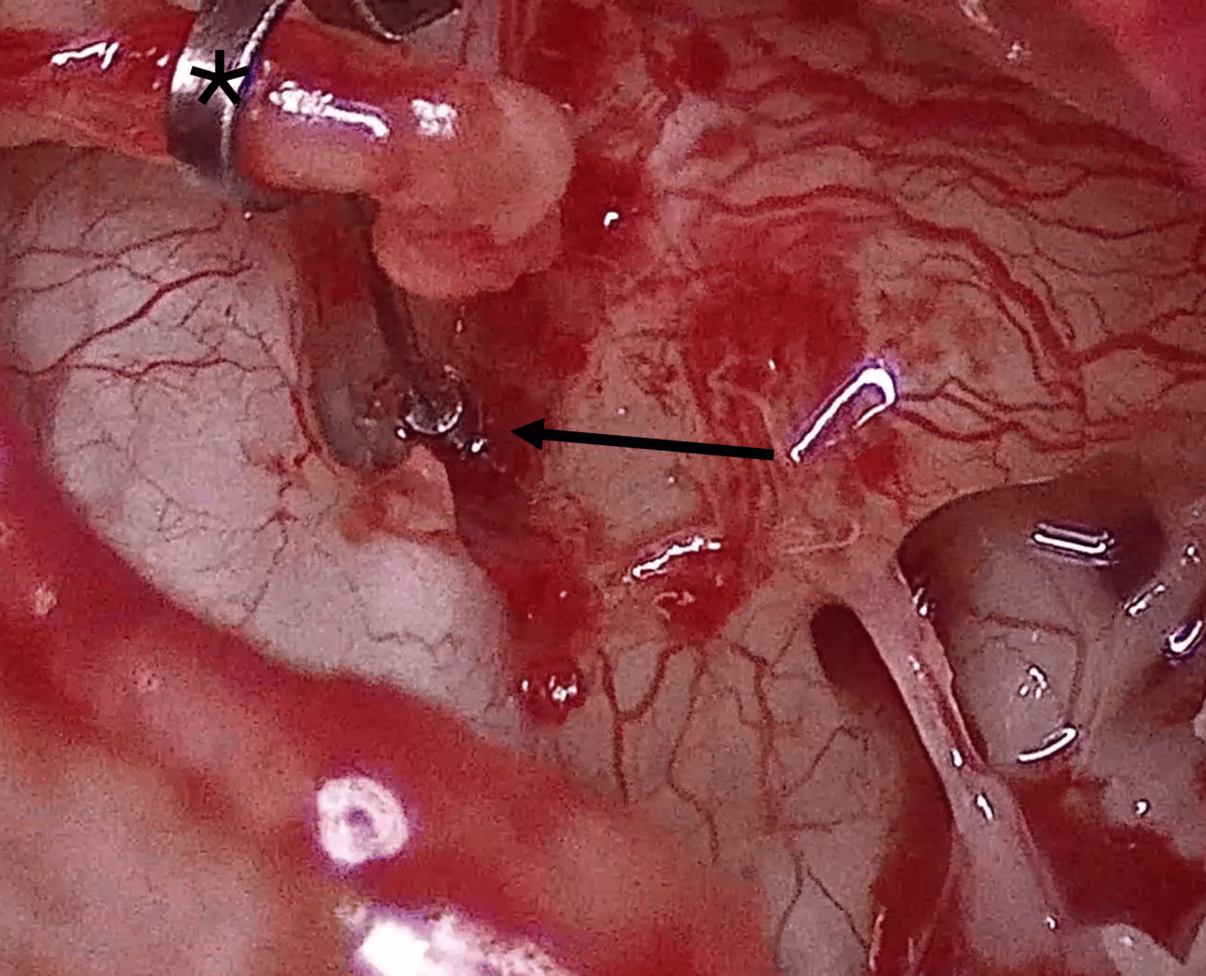
Placement of a supratitanium piston (*) with its tip (black arrow) inserted into the platinotomy

**Figure 8:**
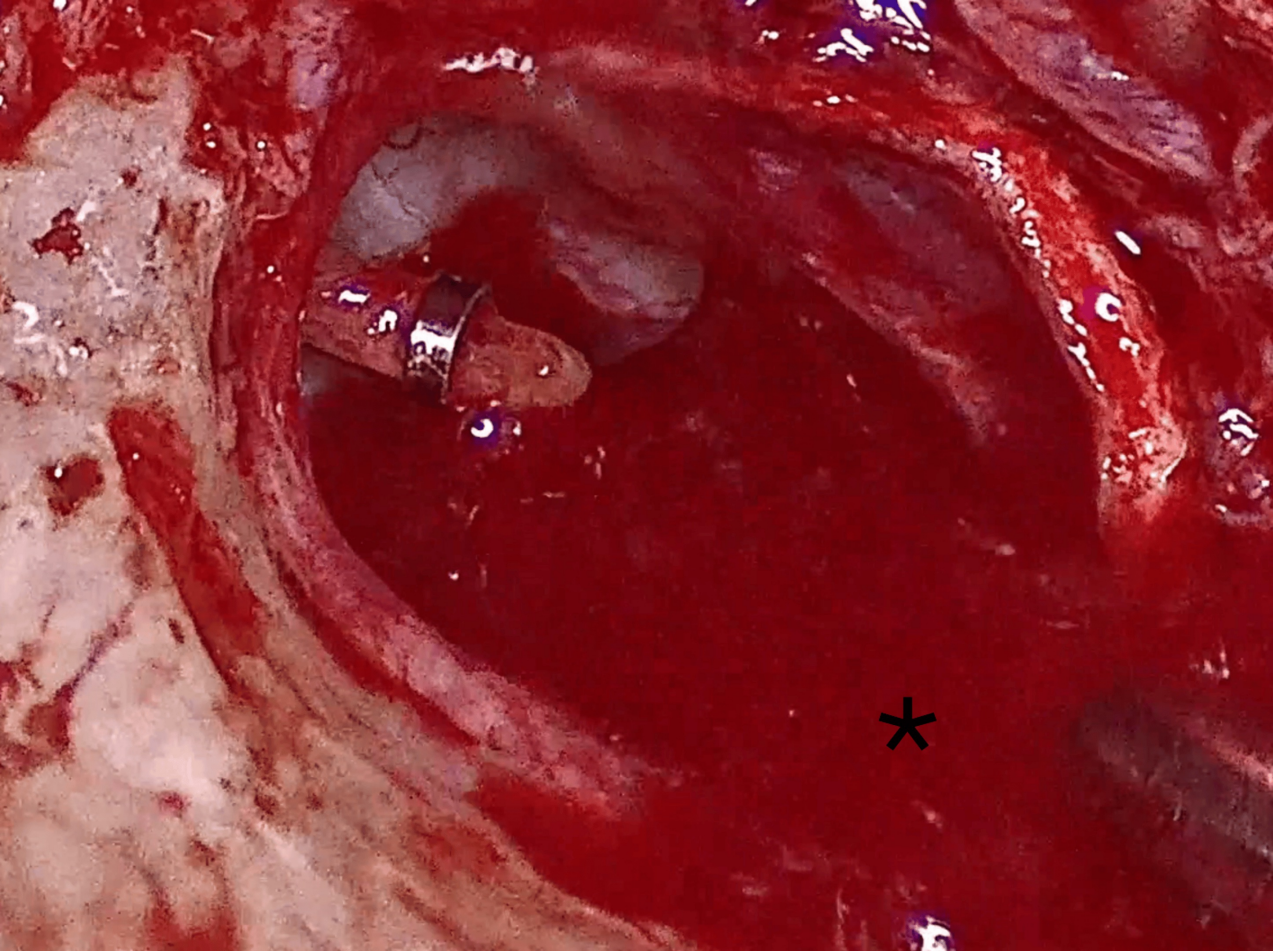
Blood drop (*) placed in the middle ear to secure the prosthesis

The distance from the footplate to the long process of the incus measured 4.5 mm (Figure 6). A super-titanium piston prosthesis was trimmed to the measured length and gently crimped onto the incus (Figure 7). Mobility testing confirmed satisfactory function. A small drop of blood was placed to stabilize the prosthesis (Figure 8), and the tympanomeatal flap was repositioned.

At six weeks postoperatively, the tympanic membrane was intact, the prosthesis remained in a stable position, and pure-tone audiometry showed closure of the ABG to within 10 dB, confirming a favorable short-term anatomical and functional outcome.

## Discussion

Current knowledge

Endoscopic stapes surgery has gained increasing acceptance as a valid alternative to the traditional microscopic approach. Its transcanal access provides a panoramic view of the middle ear while often eliminating the need for canaloplasty or wide exposure. A 2021 systematic review involving 361 patients reported a postoperative ABG ≤10 dB in 71% of cases and ≤20 dB in 97%, with complication rates comparable to microscopic techniques [1]. Similar findings were confirmed in another meta-analysis, which also highlighted a lower incidence of chorda tympani manipulation and postoperative dysgeusia in the endoscopic group [2].

Reverse stapedotomy, in which the footplate fenestration precedes removal of the stapes superstructure, offers theoretical advantages. By keeping the stapes in place during the platinotomy, the risk of footplate mobilization or floating footplate displacement is reduced. Some authors also suggest this sequence may improve footplate stability and reduce the risk of perilymph gusher [3]. Although evidence remains limited, comparative studies have shown similar audiometric outcomes between reverse and conventional techniques [4].

The manual microtrephine, used here for a 0.6 mm platinotomy, avoids the vibration and heat generation associated with a microdrill, thereby reducing the risk of inner ear trauma. Bailey et al. were among the first to promote this instrument, which remains considered safe and effective [5].

Contributions of this report

What distinguishes the present case is the integration of CT-based 3D virtual endoscopy into preoperative planning. This simulation anticipated limited visibility of the stapes suprastructure through the external auditory canal and guided the targeted creation of a Rosen notch to optimize exposure. While the use of virtual endoscopy in otologic surgery is still uncommon, feasibility studies have suggested its value for spatial planning and education [6]. To our knowledge, its direct influence on intraoperative decision-making in reverse endoscopic stapedotomy has not been previously detailed.

In addition to demonstrating the technical workflow, this report provides short-term postoperative data: at six weeks, the tympanic membrane remained intact, the prosthesis position was stable, and the ABG had closed to within 10 dB. These findings confirm both the anatomical integrity and functional improvement, endorsing the safety and feasibility of the approach.

Limitations

This report involves a single illustrative case, and long-term follow-up is not yet available. Therefore, while the short-term results are encouraging, they should be interpreted with caution. Future studies, including a larger patient cohort and extended follow-up, are required to assess the reproducibility and durability of outcomes. Finally, the inherent limitations of endoscopic ear surgery - one-handed technique, lack of depth perception, potential visual obstruction from bleeding, and heat from the light source - must be acknowledged. Nevertheless, recent literature indicates that, with adequate training, endoscopic stapedotomy can achieve surgical times, complication rates, and hearing outcomes equivalent to the conventional microscopic approach [7].

## Conclusions

This updated technical video report illustrates a reproducible method for endoscopic reverse stapedotomy. The key takeaway is that incorporating CT-based 3D virtual endoscopy into the preoperative workflow can refine surgical planning by anticipating exposure limitations and guiding intraoperative decisions such as Rosen notch creation. In this case, the combination of targeted bony removal with a manual microtrephine platinotomy resulted in a favorable short-term outcome, with anatomical integrity and closure of the ABG to within 10 dB at six weeks. While further studies are needed to confirm its impact on long-term results, this report highlights the potential of virtual endoscopy as a valuable adjunct in stapes surgery.
